# Effect of pumpkin seed addition on sensory, antioxidant, and oxidative kinetic properties of pistachio spread

**DOI:** 10.1016/j.fochx.2026.104561

**Published:** 2026-10-14

**Authors:** Arian Nahalkar, Mostafa Shahidi-Noghabi, Sara Naji-Tabasi

**Affiliations:** a Department of Food Nanotechnology, Research Institute of Food Science and Technology (RIFST), Mashhad, Iran; b Department of Green Technologies in Food Processing, Research Institute of Food Science and Technology (RIFST), Mashhad, Iran

**Keywords:** Pistachio spread, Pumpkin seeds, Optimization, Kinetic parameters

## Abstract

Pistachio spread (PS) is a popular nut-based product valued for its flavor and nutritional properties; however, its high lipid content makes it susceptible to oxidative deterioration during storage. This study aimed to develop and optimize a mixed pistachio–pumpkin seed spread (MPPS) and evaluate its sensory, physicochemical, textural, and oxidative stability characteristics. Pistachio kernels were roasted under different conditions, and roasting at 110 °C for 10 min was selected as the optimum treatment based on sensory evaluation. The optimized PS formulation consisted of 76% pistachio paste, 23.4% sugar, 0.1% salt, and 0.5% lecithin. Pumpkin seed paste was incorporated at different substitution levels, and the formulation containing 30% pumpkin seed paste achieved the highest overall acceptability based on sensory and color evaluations.

Pumpkin seeds contained higher protein, ash, and moisture contents than pistachios, whereas pistachios were richer in fat and carbohydrates. The incorporation of pumpkin seed paste significantly reduced peroxide formation and improved the physical stability of MPPS during storage compared with PS. Texture profile analysis indicated that PS exhibited higher cohesiveness, adhesiveness, and firmness, while MPPS showed greater gumminess and spreadability. Although firmness increased and spreadability decreased during storage in both formulations, MPPS maintained lower firmness and higher spreadability than PS. Kinetic analysis revealed that lipid oxidation in MPPS followed zero-order reaction kinetics. The calculated Ea, ∆H^++^,and ∆S^++^ values were 40.87 kJ/mol, 38.43 kJ/mol, and − 0.129 kJ/mol·K, respectively. Overall, pumpkin seed paste improved the nutritional quality, oxidative stability, and storage performance of PS while preserving desirable sensory properties.

## Highlights

• Pumpkin seed improved oxidative stability of pistachio spread.
• A 30% substitution level achieved the highest sensory acceptance.
• Pumpkin seed reduced peroxide formation during storage.
• The mixed spread showed improved spreadability and stability.
• Lipid oxidation followed zero-order reaction kinetics.

## 1 Introduction

Pistachio (*Pistacia vera L.*) is a widely valued nut crop, celebrated for its distinctive flavor, rich nutrient composition, and bioactive properties. The kernels are characterized by high levels of monounsaturated fatty acids, proteins, vitamins, minerals, and phenolic compounds, all of which contribute to their health-promoting effects and desirable sensory characteristics in food applications (Farrokhi et al., 2025). In addition to their nutritional benefits, pistachios are abundant in natural antioxidants, such as tocopherols and polyphenols, which play a crucial role in mitigating lipid oxidation and enhancing the shelf life of nut-based products (Kulik & Waszkiewicz-Robak, 2025). Among pistachio-derived products, pistachio spreads (PS) have gained increasing attention due to their versatility as functional foods and their rising demand among health-conscious consumers. Nevertheless, the oxidative stability and sensory quality of these spreads are often compromised during storage, largely due to lipid peroxidation, Maillard reactions, and moisture-induced textural changes.

To overcome these challenges, the inclusion of complementary ingredients with functional properties has emerged as a promising strategy. Pumpkin seeds (*Cucurbita pepo L.*) represent a particularly suitable candidate for this purpose. Sharing a similar green hue with pistachios and a mild nutty flavor, pumpkin seeds are rich in proteins, unsaturated fatty acids, minerals, and phenolic compounds, which collectively enhance both the nutritional and functional profiles of nut-based spreads (Adsul & Madkaikar, 2021; Dobón-Suárez et al., 2025). Beyond their antioxidant capacity, pumpkin seeds contain bioactive compounds with emulsifying and stabilizing properties, contributing to the physical stability and desirable texture of spreads (Nishat et al., 2024). Therefore, the synergistic incorporation of pumpkin seeds into PS is anticipated to improve oxidative stability, sensory acceptance, and textural performance, while also offering a more cost-effective and nutritionally enriched formulation.

Prior studies on nut and seed-based spreads have primarily examined processing parameters, antioxidant activity, and sensory evaluation. (Farrokhi et al., 2025), demonstrated that natural antioxidants derived from pistachio hull extracts significantly enhanced oxidative stability in PS, highlighting the potential of functional additives for prolonging shelf life. Similarly, research on pumpkin seed flour and oil has shown improvements in antioxidant activity, emulsification capacity, and sensory attributes when incorporated into spreads and bakery products (BRAHIMI Meriem and K., 2025; Dobón-Suárez et al., 2025). He et al. (2025) reported that the addition of seed-derived antioxidants in cottonseed- and pistachio-based spreads delayed lipid oxidation without adversely affecting sensory quality (He et al., 2025). Sustainability-focused investigations also indicate that valorizing seed by-products, such as pumpkin and pepper seeds, not only enhances functional properties but also contributes to waste reduction and promotes circular economy practices within the agri-food sector (Lankanayaka et al., 2025).

Despite these promising findings, there remains a notable research gap regarding the systematic optimization of PS enriched with pumpkin seeds, particularly in terms of sensory evaluation, textural properties, and oxidative kinetics. While individual studies have explored the antioxidant potential of seed additions or plant extracts, comprehensive investigations on the combined effects of pistachio and pumpkin seeds on physicochemical, sensory, and kinetic characteristics are limited. Moreover, quantitative assessments using kinetic parameters, including reaction order, activation energy, and Gibbs free energy, are essential for accurately predicting shelf life and processing stability under varying storage conditions (Fadda et al., 2022; Nishat et al., 2024).

However, important knowledge gaps remain. Most previous studies have focused on either the nutritional properties of pumpkin seeds or the antioxidant effects of individual plant extracts, whereas little attention has been paid to the combined optimization of pistachio–pumpkin seed spreads. In particular, no comprehensive study has simultaneously optimized formulation composition, evaluated sensory and instrumental quality, and characterized the oxidative behavior of such spreads through kinetic and thermodynamic analyses. Consequently, the effects of pumpkin seed incorporation on peroxide formation, texture evolution, physical stability, and oxidation kinetics during storage remain insufficiently understood.

The pumpkin seed substitution levels evaluated in this study were selected to provide a gradual partial replacement of pistachio paste while preserving the characteristic sensory properties of PS. The investigated range was chosen based on preliminary formulation trials and technological considerations to ensure acceptable texture, color, spreadability, and consumer acceptability while allowing the functional contribution of pumpkin seed paste to be effectively assessed. The optimum substitution level was subsequently determined using a combination of sensory evaluation, instrumental color analysis, and mixture-design optimization.

Therefore, the objective of this study was to optimize the formulation of PS enriched with pumpkin seed paste using mixture design methodology and to evaluate the effects of pumpkin seed incorporation on sensory attributes, physicochemical properties, texture profile, physical stability, antioxidant activity, and lipid oxidation kinetics during storage. Unlike previous studies, the present work integrates formulation optimization with kinetic and thermodynamic modeling of oxidation, providing a comprehensive understanding of the mechanisms governing the stability of mixed pistachio–pumpkin seed spreads. These findings provide valuable information for the development of nutritionally enriched, shelf-stable, and economically sustainable plant-based spreads.

## 2 Materials and methods

### 2.1 Raw materials

Dried pistachio nuts (*Pistacia vera* L., early-maturing cultivar ‘Fandoghi’) and pumpkin seeds (*Cucurbita pepo* var. *styriaca*) were obtained from the Agricultural Jihad Research Station, Kerman, Iran. The seeds were stored in breathable cotton bags at 10 °C until further use.

The pistachio nuts were manually shelled, and the kernels were separated. Subsequently, the kernels were transferred into polyethylene bags and stored under refrigerated conditions at 5 °C until analysis. All chemicals used in this study were of analytical grade and were purchased from Merck (Germany), unless otherwise specified.

### 2.2 Methods

#### 2.2.1 Determination of physicochemical properties of pistachio and pumpkin kernels

##### 2.2.1.1 Determination of protein content

The protein content of the kernel samples was determined using the micro-Kjeldahl method according to (Khosroshahi Asl, 1997). The analysis was performed using a micro-Kjeldahl apparatus (ASCO-ASU-500Sl, Iran).

##### 2.2.1.2 Determination of fat content

The fat content of the kernel samples was determined using the Soxhlet extraction method (Parvaneh, 1992). Briefly, about 5.0 g of finely ground sample was extracted with petroleum ether for 8 h. The solvent was evaporated, and the remaining extract was dried and weighed. Fat content (%) was calculated based on the difference in flask weight before and after extraction relative to the sample weight.

##### 2.2.1.3 Determination of ash content

The ash content was determined according to Parvaneh (1992). Approximately 5.0 g of ground sample was first charred and then incinerated in a muffle furnace at 520 °C for 8 h until a light gray/white ash was obtained. The crucible was cooled in a desiccator and reweighed, and ash content was calculated based on the weight difference before and after ashing .Ash content was calculated as a percentage of dry weight using the following equation:

(2-1) $$\mathrm{Ash}\;\left(\%\right)=\frac{\left(M_{2}-M_{1}\right)\times 100}{M_{0}}\;$$

In this equation, M₀ represents the initial weight of the sample before ashing, expressed in grams (g). M₁ denotes the weight of the empty crucible with its lid prior to placing the sample (g), while M₂ refers to the weight of the crucible with the lid and the remaining ash after the incineration process (g). These parameters are used to determine the ash content of the sample by calculating the weight of the inorganic residue relative to the original sample weight.

##### 2.2.1.4 Determination of moisture content

Moisture content was determined according to the Iranian National Standard No. 5693 using the oven-drying method at 70 °C (Kamangar et al., 1975). Approximately 5.0 g of the sample was accurately weighed (m₁) and placed in a drying oven at 70 °C until a constant weight was achieved. After drying, the samples were removed from the oven, cooled in a desiccator to room temperature, and weighed again (m₂). Moisture content (H) was calculated using the following equation:

(2-2) $$H\left(\%\right)=\frac{\left(m_{1}-m_{2}\right)}{m_{1}}\times 100\;$$

where, m₁ represents the initial weight of the sample before drying, expressed in grams (g), while m₂ denotes the weight of the sample after oven drying (g). The difference between these two values corresponds to the amount of moisture removed during drying and is used to calculate the moisture content of the sample as a percentage of its original weight.

##### 2.2.1.5 Determination of total sugar content

Total sugar content was determined using the Fehling titration method (Zhang & Chen, 2026). Briefly, 2.0 g of sample was extracted in a 100 mL volumetric flask, clarified, and hydrolyzed with 5 mL HCl at 70 °C for 10 min. After neutralization, the solution was diluted to 100 mL. For titration, 25 mL of the prepared extract was reacted with Fehling's solution and titrated at the boiling point using methylene blue as the indicator. The endpoint was identified by the disappearance of the blue color and the formation of a brick-red precipitate. Total sugar content was then calculated based on the volume consumed using the following equation:

(2-3) $$\text{Total sugar}\;\left(g\;\mathrm{per}\;100\;g\;\text{sample}\right)=\frac{\text{Fehling factor}\times 100\times 100\times 100}{\text{Volume of sugar solution consumed}\times \text{Sample weight}\times 25}\;$$

In this equation, the Fehling factor represents the standardization factor of the Fehling solution, which is used to convert the titration result into the corresponding sugar concentration. The volume of sugar solution consumed refers to the volume (mL) of the sample solution used during titration to reach the endpoint. The sample weight denotes the initial weight of the analyzed sample (g), and 25 mL represents the aliquot volume taken for hydrolysis prior to titration. These parameters are used collectively to calculate the sugar content of the sample based on the Fehling titration method.

#### 2.2.2 Optimization of roasting temperature and time for pistachio and pumpkin seeds

The roasting temperature and time were optimized using 100 g samples of pumpkin seeds. Roasting was carried out in a laboratory oven at four different temperatures (90, 110, 130, and 150 °C) and three different time intervals (10, 20, and 30 min). Following roasting, the samples were evaluated to determine the optimal processing conditions. Selection of the best temperature–time combination was based on sensory evaluation conducted by a trained panel, as well as instrumental color measurements. Sensory attributes were analyzed, and the treatment showing the highest overall quality scores was selected as the optimal roasting condition.

Roasting of pistachio kernels was carried out based on the optimal conditions reported by Shaker Ardakani et al. Pistachio kernels were placed in a single layer on Pyrex Petri dishes (9 cm in diameter) and were roasted in a convection oven (Memmert, UNB 500) at 130 °C for 30 min (Shakerardekani et al., 2011).

#### 2.2.3 Optimization of PS formulation

A mixture design was employed to optimize the formulation, in which the component proportions were constrained to sum to a constant total. The experimental runs were generated using the D-optimal criterion. Some mixture formulations were replicated within the experimental design to provide an estimate of pure experimental error. These replicated runs were used to assess the reproducibility of the experimental responses and to provide an independent estimate of pure error for the analysis of variance and lack-of-fit assessment.

For the preparation of the PS, pistachios roasted under the optimal temperature–time conditions determined in the previous stage were ground using a high-power mechanical grinder (Arman Khorad Toos, Iran). Powdered sugar (5–25%), salt (0.1–0.5%), and lecithin (0.1–0.5%) were added to the ground pistachio according to a mixture design. The blending process was carried out using a semi-industrial grinder (Toos Shekan Khorasan, Iran) until a uniform and homogeneous mixture was obtained. The resulting paste-like mixture was then transferred to a ball mill and processed for 60 min at ambient temperature until a smooth, creamy, and homogeneous texture was achieved.

##### 2.2.3.1 Sensory evaluation of samples with different pumpkin seed paste levels

Sensory evaluation was conducted using a panel of 15 assessors recruited from students, staff members, and laboratory experts at the Research Institute of Food Science and Technology (RIFST), Mashhad, Iran. The panel consisted of 9 female and 6 male assessors aged 25–35 years. All assessors had some prior familiarity with sensory evaluation procedures. Before participation, the assessors underwent preliminary screening based on self-reported information to ensure that they had no current illness or known impairment of taste or olfactory function. Their use of tobacco products and relevant medications was also assessed based on self-reported information. Participation was voluntary, and all assessors were informed that the samples were plant-based spreads and provided informed consent before the sensory evaluation.

The assessors participated in two training sessions before the formal sensory evaluation. The first session was conducted two days before the sensory test and focused on explaining the purpose of the evaluation, the important sensory characteristics of the product, and the attributes included in the sensory evaluation form. During the second session, conducted immediately before the formal evaluation, the scoring procedure and comparative evaluation of coded samples were explained and practiced. The meaning of the five-point scale was clearly explained to all assessors, with scores of 1 and 5 representing unacceptable and excellent sensory quality, respectively. The assessors were also instructed to allow an appropriate interval between successive samples and to cleanse the palate between samples to minimize carry-over effects.

The optimized PS was used as the control formulation, and pumpkin seed paste was incorporated at different substitution levels to prepare mixed pistachio–pumpkin seed spread (MPPS) samples. For sensory evaluation, each sample was assigned a randomly generated three-digit code. The samples were arranged and presented in a randomized order to minimize potential order effects. The assessors were blinded to the formulation composition and were not informed of the percentage of pumpkin seed paste in individual samples.

Sensory evaluation was conducted in a dedicated sensory evaluation room maintained at 25 °C under artificial lighting and free from extraneous odors. Assessors were instructed not to communicate with one another during the evaluation. Approximately 15 g of each sample was presented to each assessor in a closed polyethylene container. Samples were prepared at ambient temperature and maintained under ambient conditions in closed polyethylene containers until evaluation. Each sensory evaluation was completed in a single session, and each assessor evaluated each sample once. Water was provided between samples for palate cleansing and reduction of carry-over effects.

The samples were evaluated for color, odor, flavor, taste, and overall acceptance using a five-point sensory acceptance scale, where 1 indicated unacceptable and 5 indicated excellent sensory quality. The same panel of 15 assessors was used throughout all sensory evaluations conducted in the study.

##### 2.2.3.2 Color measurement of samples with different pumpkin paste levels

The color of the spread samples containing varying proportions of pumpkin seed paste was measured using a HunterLab ColorFlex® (0/45 model, Hunter Associates Laboratory, USA). The instrument was calibrated according to the provided standards prior to measurement. Finely ground samples were placed in a glass sample holder positioned in the designated area of the device, and the color parameters L*, a*, and b* were recorded for each sample (Afoakwa et al., 2008).

#### 2.2.4 Quality evaluation of optimized sample

For the evaluation of quality changes during storage, including peroxide value and other physicochemical and quality parameters, separate samples were prepared for each sampling time. The samples were filled into clean, tightly closed glass containers. To minimize the effect of headspace oxygen on oxidation, the containers were filled as completely as practicable, leaving only a minimal headspace. Separate containers were assigned to each sampling time to avoid repeated opening and closing of the same container, thereby minimizing additional exposure of the samples to atmospheric oxygen during storage and measurement. The sealed samples were stored in an oven at the designated storage temperatures until the corresponding sampling time, after which the appropriate analyses were performed.

##### 2.2.4.1 Oxidative stability

Oxidative stability was evaluated by measuring the peroxide value in triplicate for the optimized MPPS and a control pistachio spread sample. Briefly, 3.0 g of extracted oil was dissolved in an acetic acid–chloroform mixture (30 mL), followed by the addition of 0.5 mL of saturated potassium iodide and reaction in the dark. The released iodine was titrated with 0.001 N sodium thiosulfate using starch as an indicator, and the peroxide value was determined from the titration result. The peroxide value was calculated using the following formula (Hosseini, 1994):

(2-4) $$\text{Peroxide value}\;\left(\mathrm{meq}\;O_{2}/\mathrm{kg}\;\text{oil}\right)=\frac{\text{Volume of}\;{\mathrm{Na}}_{2}S_{2}O_{3}\times 0.001\times 100}{\text{Sample weight}\;\left(g\right)}\;$$

Acid value was determined in triplicate for the extracted oil samples. Briefly, 5.0 g of oil was dissolved in 20 mL ethanol, and 2–3 drops of phenolphthalein were added. The solution was then titrated with 0.1 N KOH until a persistent faint pink endpoint was reached. The acid value, expressed as the percentage of free fatty acids based on oleic acid, was calculated using the following equation (Haddad Khodaparast et al., 2005):

(2-5) $$\text{Acid value}\;\left(\%\right)=\frac{\text{Volume of}\;\mathrm{KOH}\;\left(\mathrm{mL}\right)\times \text{Normality of}\;\mathrm{KOH}\;\left(N\right)\times 1.65}{\text{Sample weight}\;\left(g\right)}\;$$

##### 2.2.4.2 Physical stability

The oil separation stability of pistachio spread samples was determined using a modified centrifugation method based on the procedure reported for pistachio spreads by Shakerardekani and co-workers. Approximately 15 g of each spread sample was accurately weighed into centrifuge tubes and heated in a water bath at 80 °C for 30 min to facilitate oil mobility within the matrix. The samples were subsequently cooled under running water for 15 min and centrifuged at 3000 ×*g* for 15 min. After centrifugation, the separated oil layer was carefully removed using a Pasteur pipette and weighed. The oil separation rate (OSR) was calculated as the percentage of separated oil relative to the initial sample weight according to Eq. (2–6).

(2-3) $$\;\mathrm{OSR}\;\left(g/100\;g\right)=\left(m_{1}/m_{i}\right)\times 100$$

This method was used to evaluate the colloidal stability of the spread and its resistance to oil migration (Kılıç et al., 2025).

##### 2.2.4.3 Texture analysis

The texture properties of pistachio spreads were evaluated at 25 °C using a TA.XT Plus texture analyzer (Stable Micro Systems Ltd., Surrey, UK). Back-extrusion and Texture Profile Analysis (TPA) tests were carried out to characterize the structural properties of the samples. The spreads were transferred into the standard back-extrusion container at a constant height and the sample surface was leveled prior to testing. Samples were allowed to rest for 2 min at room temperature before analysis. Test conditions included a 35 mm cylindrical probe, a test speed of 0.5 mm/s, penetration distance of 5 mm, sample height of 3 cm, and container inner diameter of 4.9 cm. Firmness, hardness, adhesiveness, cohesiveness, resilience, and elasticity were determined from the resulting force–time curves.

A penetration test was performed to evaluate the consistency and firmness of the spread samples. Approximately 200 g of each sample was placed in a cylindrical container and analyzed using a flat-ended cylindrical probe (0.48 cm diameter). The probe penetrated 1 cm into the sample at a constant speed of 200 mm/min, and the maximum penetration force was recorded as an indicator of spread consistency and structural strength (Hadinezhad et al., 2014; Shakerardekani et al., 2013).

##### 2.2.4.4 Determination of water activity

Water activity ($a_{w}$) was measured using a Novasina Sprint water activity meter (Model TH 500, Novasina, Switzerland).

##### 2.2.4.5 Rheological properties

###### 2.2.4.5.1 Time-independent rheological properties

The effect of shear rate on the rheological behavior of the spread samples was evaluated over a shear rate range of 1–300 s^−1^ in both forward and backward directions. Due to the non-Newtonian behavior of the samples, the shear stress–shear rate data were fitted to the following rheological model to describe flow behavior (Steffe, 1996):

(2-7) $$\tau =k_{p}\;{\dot{\gamma }}^{n_{p}}\;$$

In this equation, τ represents the shear stress (Pa) applied to the sample, while γ˙ denotes the shear rate (s^−1^), indicating the rate at which the sample is deformed under shear. The parameter kₚ, known as the consistency coefficient (Pa·sⁿ), reflects the viscosity level or thickness of the material, with higher values indicating greater resistance to flow. The nₚ, or flow behavior index (dimensionless), describes the type of flow behavior; values less than 1 indicate shear-thinning behavior, values equal to 1 correspond to Newtonian flow, and values greater than 1 represent shear-thickening behavior.

###### 2.2.4.5.2 Time-dependent rheological properties

To evaluate the time-dependent rheological behavior, the spread samples were subjected to a constant shear rate of 50 s^−1^, which corresponds to the effective shear rate during oral sensory perception (Bourne, 2002). Shear stress (τ) was recorded as a function of time (t) until a steady-state stress was reached. The time-dependent flow behavior of the samples was then modeled using first-order stress decay with a non-zero equilibrium stress (Steffe, 1996):

(2-8) $$\;\tau \left(t\right)=\tau_{\mathrm{eq}}+\left(\tau_{0}-\tau_{\mathrm{eq}}\right)\;e^{-\mathrm{kt}}\;$$

In this equation, initial shear stress (τ₀), which represents the stress required to initiate flow in the undisturbed structure, the equilibrium shear stress ($\tau_{\mathrm{eq}}$), indicating the residual stress level after structural relaxation under constant shear, and the structure breakdown constant (k), which quantifies the rate at which the internal network deteriorates over time under applied stress (s^−1^). These parameters collectively characterize the spread's thixotropic behavior, providing insights into how the product flows under force while partially recovering its structure once the stress is removed.

##### 2.2.4.6 DPPH radical scavenging assay

The DPPH radical scavenging activity was determined according to the method described by (Gulcin & Alwasel, 2023). Briefly, a methanolic solution of DPPH (0.1 mM) was prepared, and a defined volume of the sample extract at different concentrations was mixed with the DPPH solution. The mixture was incubated in the dark at room temperature for an appropriate period (typically 30 min to several hours, depending on reaction kinetics). The decrease in absorbance was then measured at 517 nm using a spectrophotometer. Radical scavenging activity was calculated as the percentage inhibition of DPPH based on the difference between the absorbance of the control and the sample.

##### 2.2.4.7 Determination of oxidation kinetic parameters

The radical oxidation mechanism and hydroperoxide formation in pistachio oil represent the most destructive reactions, leading to product quality deterioration during storage. Changes in peroxide values over the storage period are commonly used to determine the degree of oxidation and to estimate the shelf life of PS. The general kinetic equation for lipid peroxide formation can be expressed as:

(2-9) $$\;\frac{\mathrm{dC}}{\mathrm{dt}}=k{\left[C\right]}^{n}\;$$

In the study of lipid oxidation kinetics, the formation of peroxides during storage is described by the parameter C, representing the peroxide concentration (expressed in mol/L or meq/kg) accumulated over time. The reaction rate is governed by the rate constant k, which quantifies the speed of peroxide generation, while the reaction order n characterizes the dependency of the rate on the concentration of reactive species. tdenotes the storage time (in days or hours) during which oxidation occurs. These parameters collectively define the kinetic model used to predict oxidative changes in fat-rich foods and to evaluate shelf-life stability under different storage conditions.

Different kinetic models (zero-, first-, and second-order) can be applied to describe the increase in peroxide values over time and determine the reaction order of oxidation. The best-fitting model was selected based on the coefficient of determination (R^2^).

For the zero-order model, peroxide value (PV) was described as a function of storage time according to:

(2-10) PVₜ=PV₀+k₀t

where PVₜ is the peroxide value at time t, PV₀ is the initial peroxide value, k₀ is the zero-order reaction rate constant (meq O₂ kg^−1^ day^−1^), and t is storage time (day).

To quantify the temperature-dependent oxidative stability, the time required for a 50% increase in peroxide value relative to the initial value was calculated. The endpoint was defined as PV_endpoint = 1.5PV₀, and the corresponding time (t₅₀) was calculated as:

(2-11) $$t₅₀=\left(\mathrm{PV}\_\text{endpoint}-\mathrm{PV}₀\right)/k₀=\mathrm{PV}₀/\left(2\;k₀\right)$$

To further investigate the mechanism of peroxide formation during storage, transition state theory, along with the thermodynamic equations proposed by Eyring and Polanyi, can be applied (Vyazovkin, 2024). The relationships between the reaction rate constant (k) and thermodynamic parameters are given as:

(2-12) $$\Delta G^{‡}=\Delta H^{‡}-T\Delta S^{‡}\;$$

(2-13) $$\;k=\frac{k_{B}T}{h}\exp\left(-\frac{\Delta H^{‡}}{\mathrm{RT}}+\frac{\Delta S^{‡}}{R}\right)\;$$

Where:

• $k_{B}$ = Boltzmann constant (1.3806488 × 10^−23^ J/K)
• h = Planck constant (6.6261 × 10^−34^ J·s)
• T = absolute temperature (K)
• R = universal gas constant (8.314 J/mol·K)
• $N_{A}$ = Avogadro number (6.022 × 10^23^ mol^−1^)
• Δ$G^{‡}$ = Gibbs free energy of activation
• Δ$H^{‡}$ = enthalpy of activation
• Δ$S^{‡}$ = entropy of activation

These equations allow the estimation of both the kinetic and thermodynamic parameters of lipid oxidation in PS during storage.

To obtain the linearized form of the Eyring–Polanyi equation, the natural logarithm of the ratio of the reaction rate constant to temperature can be used:

(2-14) $$\;\ln\left(\frac{k}{T}\right)=-\frac{\Delta H^{‡}}{R}\left(\frac{1}{T}\right)+\ln\left(\frac{k_{B}}{h}\right)+\frac{\Delta S^{‡}}{R}\;$$

By plotting $\ln\left(k/T\right)$versus the reciprocal of absolute temperature $\left(1/T\right)$, a straight line is obtained. From the slope and intercept of this line, the thermodynamic activation parameters can be determined. The slope of the line is equal to $-\Delta H^{‡}/R$, from which the enthalpy of activation ($\Delta H^{‡}$) can be calculated. The intercept corresponds to:

(2-15) $$\;\;\ln\left(\frac{k_{B}}{h}\right)+\frac{\Delta S^{‡}}{R}\;$$

from which the entropy of activation ($\Delta S^{‡}$) can be determined.

Thus, linear regression of the Eyring plot provides a quantitative estimation of the activation enthalpy and entropy associated with the lipid oxidation reaction during storage.

Since the storage temperature affects the zero-order reaction rate constant (K₀), the Arrhenius equation can be expressed as follows:

(2-16) $$K=A\;\exp\left(-\frac{E_{a}}{\mathrm{RT}}\right)\;$$

where *K* is the reaction rate constant, *A* is the pre-exponential factor, *Eₐ* is the activation energy (J/mol), *R* is the universal gas constant (8.314 J·mol^−1^·K^−1^), and *T* is the absolute temperature (K).

The activation energy (Ea) was calculated from the slope of the linear regression of ln k₀ versus 1/T using the Arrhenius equation.

#### 2.2.5 Statistical analysis

The PS formulation was optimized using a D-optimal constrained mixture design in Design-Expert version 12 (Stat-Ease Inc., USA). The mixture components were pistachio paste, sugar, salt, and lecithin, and the experimental runs were generated according to the D-optimality criterion. Depending on the response, linear, quadratic, or special cubic mixture models were fitted. Model adequacy was evaluated using ANOVA, model significance, lack-of-fit, R^2^, adjusted R^2^, predicted R^2^, PRESS, CV%, and adequate precision, together with the significance of individual model terms. For the physicochemical and instrumental measurements of PS, MPPS, and other comparative samples, measurements were performed in triplicate and expressed as mean ± SD. Differences among means were assessed using one-way ANOVA followed by Tukey's HSD test at a 95% confidence level (*p* < 0.05) using IBM SPSS Statistics [version 23]. Sensory scores obtained from the 15 trained panelists were analyzed separately; mean ± SD values were calculated for each attribute and formulation, and differences among formulations were evaluated using one-way ANOVA followed by Tukey's HSD test (*p* < 0.05). Rheological parameters were analyzed using SlideWrite software (version 7.01), and graphs were plotted using Microsoft Excel (version 2017).

## 3 Results and discussion

### 3.1 Physicochemical properties of PS and MPPS

The compositional differences between PS and MPPS (Table 1) indicate that replacing part of the pistachio paste with pumpkin seed paste substantially modifies the nutritional profile of the spread. MPPS was characterized by higher protein and mineral contents, reflecting the naturally protein-rich and mineral-rich composition of pumpkin seeds. These changes may improve the nutritional value of the final product while also influencing the structural properties of the spread through enhanced protein–lipid interactions. In contrast, PS contained higher amounts of fat and carbohydrates, which are primarily responsible for its higher energy density and characteristic creamy mouthfeel. The slightly higher moisture content observed in MPPS may affect water distribution within the matrix and should therefore be considered when evaluating storage stability. Overall, the results suggest that pumpkin seed incorporation not only improves the nutritional quality of the formulation but may also influence its physicochemical functionality, including texture and oxidative behavior, due to changes in the balance between proteins, lipids, and other bioactive constituents. These compositional modifications provide a scientific basis for the subsequent evaluation of sensory properties, rheological behavior, and oxidative stability in the optimized formulations. These findings are consistent with previous investigations on nut- and seed-based spreads. Shakerardekani et al. (2013) reported that the protein and fat contents of nuts strongly affects textural properties, sensory acceptance, and oxidative stability in pistachio spreads (Ahmad Shakerardekani et al., 2013). Mousazadeh et al. (2014) demonstrated that partial substitution with high-protein seeds, such as pumpkin seeds, can enhance overall protein content while positively influencing texture and product stability (Mousazadeh et al., 2014). Emadzadeh et al. (2013) further emphasized that maintaining a proper balance among protein, fat, and moisture is essential to produce spreads with optimal quality and consumer appeal (Emadzadeh et al., 2013). Collectively, these studies support the current findings, confirming that judicious selection and proportioning of PS and MPPS can simultaneously improve nutritional value, modulate textural behavior, and maintain desirable sensory characteristics, thereby optimizing the overall functional and organoleptic quality of nut-based formulations.

**Table 1:** Physicochemical properties of PS and MPPS (g/100 g, dry weight).

Sample	Protein	Fat	Carbohydrates	Ash	Moisture
PS	19.65 ± 0.21ᵇ	53.95 ± 0.34ᵃ	18.31 ± 0.18ᵃ	2.99 ± 0.06ᵇ	2.01 ± 0.05ᵇ
MPPS	33.06 ± 0.27ᵃ	45.50 ± 0.29ᵇ	14.33 ± 0.14ᵇ	7.10 ± 0.08ᵃ	6.01 ± 0.07ᵃ

Values are presented as mean ± standard deviation (*n* = 3). Different superscript letters within the same column indicate significant differences between samples according to Tukey's multiple comparison test (*p* < 0.05).

### 3.2 Optimization of roasting temperature and time of pumpkin seeds

Table 2 summarizes the sensory evaluation of MPPS prepared under different roasting temperature–time combinations. Roasting temperature had a greater influence on sensory quality than roasting time, indicating that thermal intensity was the dominant factor governing flavor and appearance development. Moderate roasting temperatures (90–110 °C) generally produced the highest sensory acceptance, whereas excessive roasting temperatures (130–150 °C) resulted in a marked deterioration of sensory attributes. Among the evaluated treatments, roasting at 110 °C for 10 min provided the optimum balance among flavor, aroma, color, and overall acceptability and was therefore selected as the optimum roasting condition for subsequent experiments. The superior sensory quality obtained under these conditions can be attributed to controlled Maillard reactions and lipid-derived flavor generation, which produce desirable volatile compounds responsible for roasted and nutty aromas. At the same time, the relatively short roasting period minimized excessive pigment formation, lipid oxidation, and thermal degradation of volatile compounds.

**Table 2:** The comparison of sensory properties of MPPS at different roasting conditions.

Treatment	Color	Odor	Taste	Total Acceptance
90 °C – 10′	3.8ᵃ	3.3ᵃᵇ	3.5ᵃᵇ	3.5ᵃ
110 °C – 10′	3.5ᵃ	3.7ᵃ	3.8ᵃ	3.6ᵃ
130 °C – 10´	2.5ᵇ	3.0ᵇ	2.9ᵇ	2.8ᵇ
150 °C – 10´	1.6ᶜ	2.0ᶜ	1.8ᶜ	1.8ᶜ
90 °C – 20´	3.8ᵃ	3.5ᵃ	3.6ᵃ	3.5ᵃ
110 °C – 20´	3.2ᵃ	3.6ᵃ	3.8ᵃ	3.5ᵃ
130 °C – 20´	2.4ᵇ	2.8ᵇ	2.6ᵇ	2.6ᵇ
90 °C – 30´	3.5ᵃ	3.5ᵃ	3.6ᵃ	3.5ᵃ
110 °C – 30´	3.0ᵃᵇ	3.4ᵃ	3.4ᵇ	3.4ᵃ

Values are expressed as mean scores (*n* = 15). Different superscript letters (a–c) within the same column indicate statistically significant differences among roasting treatments according to one-way ANOVA followed by Tukey's multiple comparison test (*p* < 0.05). Means sharing at least one common letter are not significantly different (*p* ≥ 0.05).

Increasing either roasting temperature or roasting time adversely affected sensory quality. At temperatures above 130 °C, thermal degradation became predominant over flavor development, resulting in darker color, loss of characteristic aroma, and the formation of bitter compounds. These changes are mainly associated with excessive Maillard reactions, caramelization, degradation of unsaturated lipids, and the formation of secondary oxidation products, all of which reduce sensory acceptance. The exclusion of samples roasted at 130 and 150 °C for 30 min further confirms that excessive thermal treatment caused severe quality deterioration.

These findings demonstrate that optimization of roasting conditions is essential for maximizing the sensory quality of MPPS by balancing desirable flavor formation against undesirable thermal degradation.

For example, Peng et al. (2021) reported that roasting at moderate temperatures improved the flavor and sensory characteristics of pumpkin seeds while maintaining phenolic content and antioxidant activity (Peng et al., 2021). Similarly, Vujasinović et al. (2012) found that controlled roasting temperature and duration were essential for optimizing sensory attributes and preventing over-browning in hull-less pumpkin seeds (Vujasinović et al., 2012). Kozłowska et al. (2025) also demonstrated that moderate roasting preserves desirable aroma and taste, contributing to higher consumer acceptability (Kozłowska et al., 2025). Finally, Şen and Gökmen (2022) highlighted that Maillard reaction kinetics are highly temperature-dependent, and moderate roasting temperatures favor the development of optimal color and flavor in seeds and nuts (Şen & Gökmen, 2022). Collectively, these studies support the current observation that 110 °C for 10 min was the most effective roasting condition for MPPS, achieving a superior balance of color, taste, aroma, and overall acceptability.

### 3.3 Optimization of PS formulation components

Table 3 presents the D-optimal mixture design and the corresponding sensory and instrumental responses used to optimize the PS formulation. The results demonstrated that the proportions of pistachio, sugar, salt, and lecithin significantly influenced the sensory acceptance and textural characteristics of the product, confirming the suitability of the mixture design for formulation optimization. Rather than the effect of a single ingredient, the quality of PS was primarily governed by the interactions among formulation components. Among the evaluated ingredients, pistachio paste contributed to the characteristic flavor and color of the spread, whereas sugar improved flavor balance by masking the natural bitterness of roasted pistachio and enhancing consumer acceptability. Lecithin played a critical technological role by improving fat dispersion and reducing phase separation, thereby enhancing spreadability and producing a more homogeneous structure. These effects are attributed to the amphiphilic nature of lecithin, which reduces interfacial tension between the lipid and solid phases and strengthens emulsion stability. In contrast, formulations containing unbalanced proportions of the ingredients exhibited reduced sensory quality and less favorable textural properties, indicating that excessive amounts of individual components cannot compensate for an inadequate formulation balance. The observed variations in hardness, adhesiveness, and spreadability demonstrate that the rheological behavior of PS is highly dependent on the interactions between the continuous fat phase and the dispersed solid particles. Appropriate sugar and lecithin concentrations promoted a more stable structural network, whereas an insufficient emulsifier concentration or excessive solid content increased firmness and reduced spreadability. These findings are consistent with the behavior of concentrated fat-based dispersions, where particle packing, fat crystallization, and emulsion stability collectively determine texture and consumer perception. Overall, the D-optimal mixture design successfully identified the optimal combination of ingredients that simultaneously maximized sensory acceptance and improved instrumental quality attributes. These results highlight the importance of considering ingredient interactions rather than individual component effects when developing high-quality pistachio spreads and demonstrate the effectiveness of mixture design as a formulation optimization tool.

**Table 3:** Experimental Runs and Sensory Responses of the D-optimal Mixture Design Used for Optimization of PS Formulation.

Run	Independent variables				Responses						
	**Pistachio (%)**	**Sugar (%)**	**Salt (%)**	**Lecitin (%)**	**Color**	**Oiliness**	**Spread ability**	**Hardness**	**Adhesiveness**	**Taste**	**Total acceptance**
1	84.6	14.6	0.4	0.4	3.6	1.5	4	3.5	3.3	4.2	4
2	82.9	16.4	0.5	0.2	3.7	3.7	4	3.5	3.7	4.3	3.8
3	89.4	9.9	0.5	0.2	3.2	3.9	4.5	2.2	3.2	4.3	3.8
4	94.6	5.0	0.1	0.3	3.1	3.5	4.9	1.3	2.8	3.1	3.7
5	75.0	24.4	0.1	0.5	4.6	1	4.3	4.9	5	5	5
6	75.0	24.4	0.5	0.1	4.5	3.5	3.9	4.4	5	4.7	4.6
7	91.2	8.1	0.5	0.2	3.1	4	4.6	2	3	4	3.5
8	86.6	12.7	0.2	0.5	3.6	1.4	4	3.1	3.1	4.5	4.5
9	88.1	11.5	0.3	0.1	3.5	4.1	4.2	2.5	3.3	4.1	3.7
10	76.9	22.1	0.5	0.5	4.5	1.2	3.75	4.5	4.1	5	5
11	81.0	18. 3	0.5	0.2	3.8	3.5	3.8	3.8	3.8	4.5	3.9
12	75.0	24.4	0.5	0.1	4.6	3.7	4	4.3	4.9	4.8	4.4
13	92.8	6.2	0.5	0.5	3	2	4.5	1.7	2.9	4.1	4
14	92.8	6.2	0.5	0.5	3	2.2	4.5	1.8	2.6	4.2	4.3
15	79.4	20.4	0.1	0.1	3.89	3.8	3.5	3.9	3.9	4.7	4
16	75.0	24.4	0.3	0.3	5	1.8	4	4.7	4.7	4.7	4.6
17	76.9	22.1	0.5	0.5	4.4	1.1	3.8	4.6	4.15	5	5
18	94.6	5.0	0.1	0.3	3	3.7	4.8	1.2	2.8	3.5	3.5
19	79.4	20.4	0.1	0.1	4.1	3.9	3.5	4	3.9	4.8	4
20	94.6	5.0	0.3	0.1	3.2	5	4.8	1.1	2.5	3.2	2.5

The findings of the present study align closely with previous research on PS optimization, demonstrating the effectiveness of mixture design approaches in capturing the complex interactions among pistachio, sugar, salt, and emulsifier. A Shakerardekani (2017) emphasized that a balanced combination of pistachio, oil, and emulsifier is crucial for achieving desirable textural characteristics, spreadability, and consumer acceptance (A Shakerardekani, 2017). Similarly, Mousazadeh et al. (2014) reported that optimizing the ratios of pistachio and added oil significantly influenced hardness, spreadability, and overall acceptance, and that mixture models allowed precise prediction of both sensory and instrumental responses (Mousazadeh et al., 2014). Emadzadeh et al. (2013) further highlighted that appropriate distribution of pistachio and oil enhances textural control and improves consumer preference in low-fat formulations (Emadzadeh et al., 2013). In addition, fine-tuning ingredient proportions enables predictable modulation of spreadability, hardness, and adhesiveness, reinforcing the notion that mixture design provides a robust and reliable framework for developing pistachio spreads with optimized mechanical and sensory qualities. Collectively, these studies corroborate the present results, confirming that careful formulation through mixture design can achieve a harmonious balance of textural integrity, sensory appeal, and overall product quality (Kılıç et al., 2025; Malvano et al., 2024).

Table 4 summarizes the instrumental texture responses obtained from the mixture design for PS formulations. The results indicate that the proportions of pistachio paste, sugar, salt, and lecithin significantly affected the instrumental textural properties of the spreads., confirming that texture is governed by the interactions among formulation components rather than by the effect of a single ingredient.

**Table 4:** Experimental runs and instrumental texture responses of PS formulations generated using the D-optimal mixture design.

Run	Independent variables				Responses		
	**Pistachio (%)**	**Sugar (%)**	**Salt (%)**	**Lecitin (%)**	**Spreadability (instrumental)**	**Hardness (instrumental)**	**Adhesiveness (instrumental)**
1	84.6	14.6	0.4	0.4	0.016	0.24	0.235
2	82.9	16.4	0.5	0.2	0.018	0.265	0.245
3	89.4	9.9	0.5	0.2	0.02	0.238	0.194
4	94.6	5.0	0.1	0.3	0.035	0.21	0.154
5	75.0	24.4	0.1	0.5	0.019	0.312	0.26
6	75.0	24.4	0.5	0.1	0.014	0.286	0.27
7	91.2	8.1	0.5	0.2	0.03	0.235	0.181
8	86.6	12.7	0.2	0.5	0.016	0.245	0.185
9	88.1	11.5	0.3	0.1	0.019	0.24	0.229
10	76.9	22.1	0.5	0.5	0.013	0.29	0.25
11	81.0	18.3	0.5	0.2	0.014	0.276	0.24
12	75.0	24.4	0.5	0.1	0.017	0.284	0.265
13	92.8	6.2	0.5	0.5	0.03	0.22	0.18
14	92.8	6.2	0.5	0.5	0.025	0.23	0.161
15	79.4	20.4	0.1	0.1	0.011	0.277	0.249
16	75.0	24.4	0.3	0.3	0.015	0.31	0.255
17	76.9	22.1	0.5	0.5	0.014	0.3	0.252
18	94.6	5.0	0.1	0.3	0.034	0.2	0.16
19	79.4	20.4	0.1	0.1	0.013	0.277	0.249
20	94.6	5.0	0.3	0.1	0.034	0.2	0.152

The balance among pistachio paste, sugar, salt, and lecithin was an important factor governing the instrumental texture of the spreads. Formulations with higher pistachio proportions and correspondingly lower sugar proportions generally exhibited differences in hardness, adhesiveness, and spreadability, indicating that the balance among the mixture components influenced the structural properties of the spread. Lecithin played an important role in modifying product structure through its emulsifying properties. By reducing interfacial tension and improving fat dispersion, lecithin contributed to a more homogeneous matrix and enhanced structural stability. Nevertheless, its effectiveness depended on the overall formulation composition, indicating that emulsifier performance is strongly influenced by interactions with the fat and solid phases rather than by concentration alone. Although sugar was incorporated at relatively low levels, it contributed to the organization of the continuous phase and influenced texture through interactions with the dispersed solid particles. These effects became evident only when sugar was combined with appropriate levels of pistachio paste and lecithin, emphasizing the importance of formulation balance.

Overall, the mixture design successfully identified the relationships between ingredient composition and instrumental texture responses, demonstrating that optimization of fat distribution and emulsifier concentration is essential for producing PS with desirable spreadability, moderate hardness, and low adhesiveness. These findings provide valuable guidance for the rational formulation of nut-based spreads with improved consumer acceptability and physical stability.

The results of the present study are in strong agreement with previous research on the optimization of PS, where mixture design approaches were effectively employed to balance instrumental and sensory properties. Kılıç et al. (2025) demonstrated that carefully adjusted ingredient proportions can maintain desirable rheological, textural, and sensory characteristics (Kılıç et al., 2025). Malvano et al. (2024) found that formulation adjustments in high-protein, sugar-free pistachio spreads produced predictable changes in textural behavior, confirming the utility of mixture design for fine-tuning mechanical and sensory attributes (Malvano et al., 2024). Similarly, Emadzadeh et al. (2013). observed that altering pistachio and oil percentages in low-fat spreads allowed precise control over spreadability and firmness (Emadzadeh et al., 2013) Zahedi and Mazaheri-tehrani (2012). reported comparable findings in halva spreads, noting that mixture design effectively captured the nonlinear interactions among ingredients affecting texture (Zahedi & Mazaheri-tehrani, 2012). Finally, Marc et al. (2024) highlighted that increasing nut oil content and optimizing fat distribution can reduce hardness while enhancing adhesiveness in protein-enriched spreads (Marc et al., 2024). Collectively, these studies corroborate the present findings, confirming that second- and third-order mixture models reliably predict textural behavior and provide a robust framework for designing PS with optimal hardness, adhesiveness, and spreadability, thereby ensuring both instrumental quality and consumer satisfaction.

Linear, second-order, and third-order models (Eqs. 3–1, 3–2, and 3–3) were employed to represent the fitted values of the responses. The significance of each model was evaluated using analysis of variance (ANOVA) at a 5% significance level.

Linear model (Eq. 3–1):

$$Y=b_{1}X_{1}+b_{2}X_{2}+b_{3}X_{3}+b_{4}X_{4}$$

Second-order model (Eq. 3–2):

$$Y=b_{1}X_{1}+b_{2}X_{2}+b_{3}X_{3}+b_{4}X_{4}+b_{12}X_{1}X_{2}+b_{13}X_{1}X_{3}+b_{14}X_{1}X_{4}+b_{23}X_{2}X_{3}+b_{24}X_{2}X_{4}+b_{34}X_{3}X_{4}$$

Third-order model (Eq. 3–3):

$$\begin{array}{cc} & Y=b_{1}X_{1}+b_{2}X_{2}+b_{3}X_{3}+b_{4}X_{4}+b_{12}X_{1}X_{2}+b_{13}X_{1}X_{3}+b_{14}X_{1}X_{4}\\ & +b_{23}X_{2}X_{3}+b_{24}X_{2}X_{4}+b_{34}X_{3}X_{4}+b_{123}X_{1}X_{2}X_{3}+b_{124}X_{1}X_{2}X_{4}+b_{234}X_{2}X_{3}X_{4} \end{array}$$

In these models, Y represents the dependent variables, including color, oiliness, spreadability, hardness, adhesiveness, taste, overall acceptance, and stability. The coefficient b corresponds to the regression parameters, while X indicates the proportion of each formulation component. The selection of the most appropriate model for each response was based on the significance of the overall model, lack-of-fit test, coefficient of determination (R^2^), adjusted R^2^, predicted R^2^, and coefficient of variation (CV%). Therefore, the selected model for each response represented the best overall balance between statistical significance, goodness of fit, and predictive capability following the approach recommended by (Chicco et al., 2021). Table 5 summarizes the regression models developed to describe the effects of formulation variables on the instrumental and sensory responses of PS. The high coefficients of determination R^2^ demonstrate that the selected mixture models adequately represented the experimental data and accurately predicted the responses within the studied formulation range. The results indicate that instrumental responses, including spreadability, hardness, and adhesiveness, were satisfactorily described by quadratic models, suggesting that these properties are primarily governed by direct interactions among formulation components. In contrast, several sensory attributes required cubic models, indicating more complex and nonlinear relationships. This difference reflects the multifactorial nature of sensory perception, which depends not only on the physical properties of the product but also on the simultaneous interaction of appearance, flavor, mouthfeel, and aroma.The regression coefficients further demonstrate that the quality of PS is controlled by the combined effects of formulation ingredients rather than by individual components alone. Model for each response.

**Table 5:** Equations and statistical parameters of different responses.

	**Response**	***P*-Value of model**	**P-Value of lack of fit**	**R**^**2**^	**Adjusted R**^**2**^	**Predicted R**^**2**^	**C.V.%**
**Sensory**	**Color**	< 0.0001	0.0739	0.9659	0.9502	0.9084	3.83
	**Oiliness**	< 0.0001	0.8809	0.9962	0.9880	0.8755	4.78
	**Spread ability**	< 0.0001	0.2492	0.930	0.9833	0.8564	1.33
	**Hardness**	< 0.0001	0.3459	0.9983	0.9965	0.7751	2.45
	**Adhesiveness**	< 0.0001	0.1877	0.9809	0.9638	0.8758	4.04
	**Taste**	< 0.0001	0.1180	0.8971	0.8697	0.7995	4.87
	**Overall acceptance**	< 0.0001	0.3796	0.9851	0.9742	0.9576	2.39
	**Physical stability**	< 0.0001	0.5922	0.9997	0.9990	0.8896	0.53
**Instrumental**	**Spread ability**	< 0.0001	0.1362	0.9003	0.8737	0.8339	14.10
	**Hardness**	< 0.0001	0.1362	0.9585	0.9474	0.9127	3.16
	**Adhesiveness**	< 0.0001	0.1591	0.9704	0.9531	0.9141	4.014
	**Equations**⁎						
**Color** = 3.34 A + 4.17B − 1093.38C − 22.26D + 1092.60 AC + 1140.63 BCE + 58.34BD **Oiliness** = 11.31 A − 0.9942B + 24,286.08C + 35,653.54D + 16.30AB − 25,195.01 AC − 36,740.62 CE − 24,548.85 BCE − 36,235.57BD − 2.938E+06CD − 690.41ABC − 1681.53ABD + 3.016E+06ACD + 2.990E+06BCD **Spread ability** = 3.57 A + 4.86B − 6427.42C − 24.85D − 6.19AB + 6629.40 AC + 133.66 CE + 6517.82 BCE + 4.008E+05CD + 381.49ABC − 4.132E+05ACD − 4.106E+05BCD **Hardness** = 0.4694 A + 4.85B − 21.27C − 8814.95D + 93.84 AC + 8989.58 CE + 8993.10BD + 3.998E+05CD + 507.78ABD − 4.180E+05ACD − 4.066E+05BCD **Adhesiveness** = 2.51 A + 4.64B + 761.29C − 858.52D − 1.43AB − 753.29 AC + 903.14 CE − 755.56 BCE + 895.42BD − 1981.71CD Taste = 3.12 A + 4.98B + 35.42C + 17.94D − 43.25 BCE **Overall acceptance** = 1.83 A + 3.05B + 75.67C + 149.07D + 6.94AB − 45.87BD − 2091.35CD − 383.85ABC − 353.26ABD **Stability** = −1.75 A + 30.28B − 64,810.10C − 84,585.95D − 51.16AB + 67,145.01 AC + 87,278.39 CE + 65,441.17 BCE + 85,884.90BD + 7.608E+06CD + 2582.93ABC + 4280.93ABD − 7.819E+06ACD − 7.745E+06BCD **Spreadability (instrumental)** = 0.0347 A + 0.0142B + 0.0670C + 0.0783D − 0.0381AB **Hardness (instrumental)** = 0.2145 A + 0.2938B + 0.2198C − 0.6919D + 1.94BD **Adhesiveness (instrumental)** = 0.1809 A + 0.2357B + 1.74C − 76.18D + 0.1388AB − 3.42 AC + 75.06 CE + 79.06BD							

⁎ In these equations, A, B, C, and D represent the proportions of pistachio paste, sugar, salt, and lecithin in the formulation, respectively, while AB, AC, AD, BC, BD, and CD represent the corresponding binary interaction terms between the mixture components.

Interactions among pistachio paste, sugar, salt, and lecithin influenced both texture and sensory acceptance, emphasizing the importance of formulation balance. In particular, the interaction between the lipid phase and lecithin played a key role in determining emulsion stability and fat dispersion, which directly affected spreadability, hardness, and adhesiveness. These observations are consistent with the behavior of concentrated fat-based dispersions, where interfacial interactions and particle organization govern the rheological properties of the final product. The significance of higher-order interaction terms for several sensory responses indicates the presence of synergistic effects among formulation components. Such nonlinear interactions cannot be explained by the contribution of individual ingredients alone and highlight the necessity of applying mixture design methodology to optimize complex food formulations. Overall, the developed regression models provide a reliable tool for predicting the quality attributes of PS and demonstrate that simultaneous optimization of sensory perception and instrumental texture requires careful adjustment of ingredient proportions rather than modification of a single component. The results also confirm the suitability of response surface methodology coupled with mixture design for developing nut-based spreads with improved quality and consumer acceptability. The results of the present study are consistent with previous reports on the optimization of PS using mixture design approaches. Shakerardekani demonstrated that second-order mixture models effectively predicted both instrumental and sensory properties of pistachio spread, emphasizing that interactions among pistachio, oil, sugar, and emulsifier play a critical role in determining texture, spreadability, and overall acceptance (A Shakerardekani, 2017). Similarly, (Mousazadeh & Mousavi, 2023) reported that higher-order models provided accurate estimations of hardness, adhesiveness, and spreadability, showing strong agreement between predicted and experimental values (Mousazadeh & Mousavi, 2023). Emadzadeh et al. (2011) also confirmed that response surface methodology and polynomial models could simultaneously optimize sensory attributes and instrumental responses in pistachio spread, highlighting the importance of balancing formulation components (Emadzadeh et al., 2011). Kılıç et al. (2025) observed that third-order models offered superior fitting for complex sensory attributes, such as oiliness and overall acceptance, compared to linear or second-order models (Kılıç et al., 2025). Furthermore Malvano et al. (2024) reported that careful adjustment of ingredient proportions in high-protein, sugar-free PS led to optimized mechanical and sensory characteristics, with predicted values closely matching experimental observations (Malvano et al., 2024). Collectively, these studies corroborate the present findings, confirming that the use of second- and third-order polynomial mixture models provides reliable predictions, accurately captures nonlinear interactions among ingredients, and facilitates the development of PS with desirable textural and sensory properties.

Table 6 presents the predicted and experimentally validated responses for the optimized PS formulation obtained using the mixture design approach. The optimization aimed to maximize sensory quality, physical stability, and instrumental spreadability while minimizing oiliness. The high overall desirability value indicates that the selected formulation successfully achieved a balanced compromise among the multiple quality attributes considered during optimization.

**Table 6:** Response values at the optimized formulation obtained using the D-optimal mixture design and the corresponding confirmatory experimental results.

Response	Experimental	Predicted
Pistachio (%)	76.0	76.0
Sugar (%)	23.4	23.4
Salt (%)	0.1	0.1
Lecithin (%)	0.5	0.5
Color	4.20	4.75
Oiliness	1.50	0.99
Spreadability (Sensory)	4.60	4.30
Hardness (Sensory)	4.10	4.90
Adhesiveness (Sensory)	4.20	5.00
Total acceptance	4.50	4.96
Physical stability (%)	22.5	23.5
Spreadability (Instrumental)	0.025	0.015
Hardness (Instrumental)	0.321	0.311
Adhesiveness (Instrumental)	0.270	0.257

The optimized formulation (desirability = 0.85) was validated experimentally. The close agreement between predicted and experimental values, particularly for physical stability (22.5 vs. 23.5%), confirms the adequacy of the optimization model and the reliability of the selected formulation.

The close agreement between the predicted and experimental responses confirms the adequacy of the developed polynomial models for describing the behavior of the formulation system. Minor deviations between predicted and measured values were observed; however, these differences remained within the expected range of experimental uncertainty and sensory variability, demonstrating that the optimization model provides reliable predictions under practical conditions. The successful validation further indicates that the selected ingredient proportions effectively balanced sensory quality with desirable textural and physical characteristics, confirming the robustness of the optimization strategy. From a technological perspective, the optimized formulation provided a stable structural network with appropriate spreadability and firmness while maintaining favorable sensory acceptance. This balance can be attributed to the combined effects of pistachio paste, sugar, salt, and lecithin on fat distribution, emulsion stability, and particle interactions within the spread matrix. These findings emphasize that simultaneous optimization of multiple responses is essential for developing high-quality nut-based spreads, as improvement in one quality attribute may adversely affect another if formulation variables are optimized independently. Fig. 1 further validates the predictive performance of the optimization models by demonstrating a strong linear relationship between the predicted and experimental responses. The close distribution of the data points around the line of identity, together with the high coefficient of determination, confirms the excellent predictive capability of the developed models and indicates the absence of substantial systematic bias. This high level of agreement demonstrates that the mixture design approach accurately described the relationships between formulation variables and product responses. Overall, the validation results combining mixture design with response surface methodology is an effective and reliable tool for optimizing PS formulations. The optimized model not only accurately predicted the experimental responses but also provided a practical framework for developing nut-based spreads with improved sensory quality, physical stability, and processing performance. The findings of the present study are in agreement with previous reports on the optimization of PS using mixture design approaches. (Mousazadeh & Mousavi, 2023) demonstrated that quantitative descriptive analysis and mixture models could accurately predict both textural and sensory attributes, allowing precise optimization of spread properties (Mousazadeh & Mousavi, 2023). Similarly, (A Shakerardekani, 2017) reported that controlling particle size distribution and balancing ingredient proportions significantly improved consumer acceptance while ensuring strong alignment between sensory evaluation and instrumental measurements (A Shakerardekani, 2017). Kılıç et al. (2025) highlighted that well-designed formulations maintain desirable rheological and sensory characteristics, confirming that ingredient ratios are critical in achieving product quality (Kılıç et al., 2025). Emadzadeh et al. (2011) found that response surface methodology and polynomial models effectively optimized low-fat pistachio spreads, providing close agreement between predicted and observed sensory and instrumental responses (Emadzadeh et al., 2011). Moreover, Malvano et al. (2024) demonstrated that modifications in formulation, including protein enrichment and sugar reduction, could predictably influence both sensory perception and instrumental characteristics, further validating the utility of mixture design in pistachio-based products (Malvano et al., 2024). Collectively, these studies corroborate the present results, confirming that second- and third-order mixture models are robust tools for optimizing pistachio spreads, accurately capturing nonlinear interactions among ingredients, and achieving a desirable balance of sensory and instrumental qualities.

**Fig. 1:**
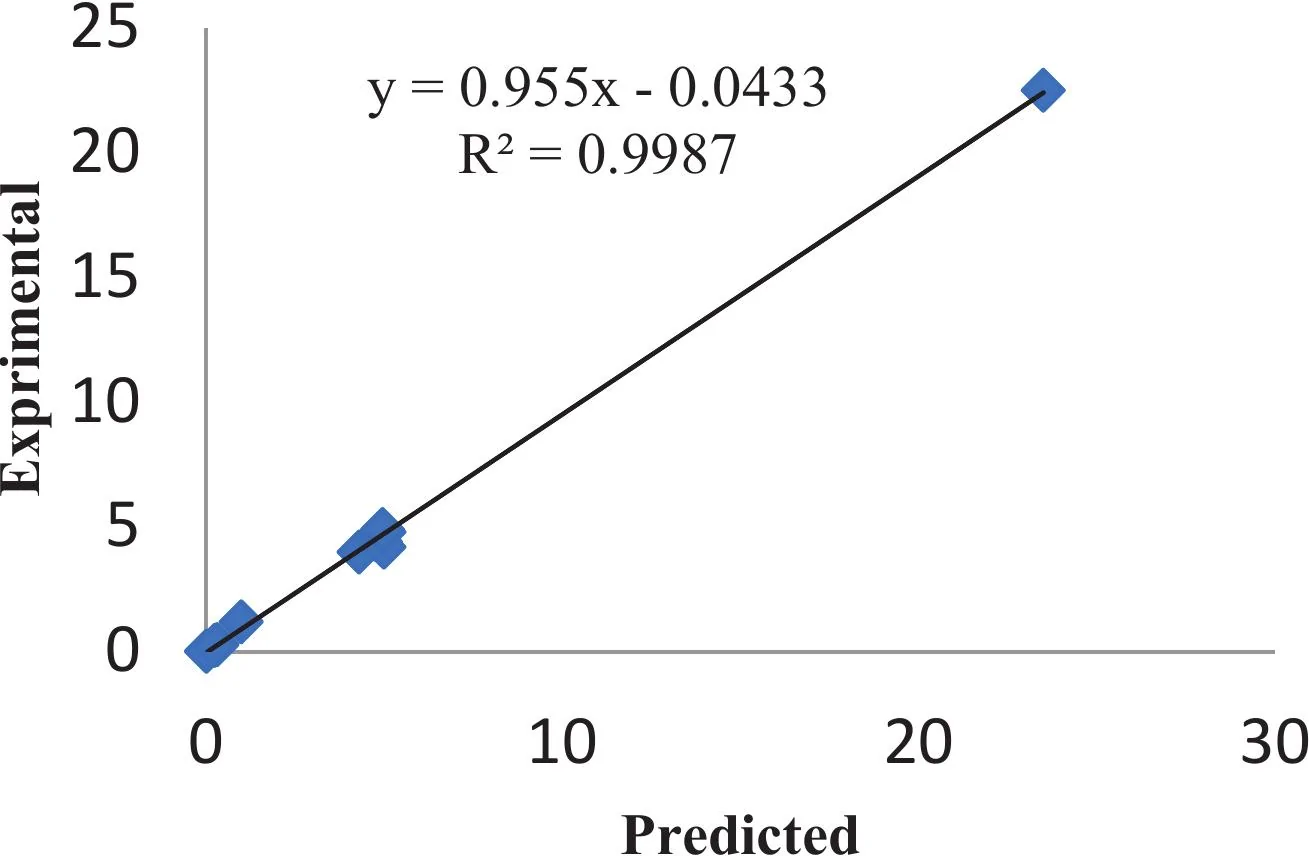
Correlation of modeled and experimental responses in the optimal formulation.

### 3.4 Determining the appropriate percentage of mixing MPPS

#### 3.4.1 Sensory properties

Table 7 summarizes the sensory evaluation of PS containing different levels of MPPS. The results demonstrate that the substitution level significantly affected the sensory responses of the trained panel, indicating that an appropriate balance between pistachio and pumpkin seed pastes is essential for maintaining desirable sensory quality. Moderate incorporation of MPPS improved the sensory profile of the product, whereas excessive substitution negatively affected consumer acceptance. The formulation containing 30% pumpkin seed paste showed the highest overall sensory acceptance among the tested substitution levels and was therefore selected for subsequent analyses. The sensory results indicated that moderate substitution provided a favorable balance between the characteristic sensory properties of pistachio spread and the sensory contribution of pumpkin seed paste, whereas higher substitution levels resulted in lower acceptance scores. At higher substitution levels, sensory acceptance declined because the flavor and aroma of pumpkin seed became increasingly dominant, masking the characteristic roasted pistachio attributes. In addition, the darker pigmentation of pumpkin seed paste reduced visual appeal, while changes in the fat–protein matrix may have influenced mouthfeel and overall eating quality. These observations indicate that sensory acceptance is determined not only by flavor intensity but also by the combined effects of appearance, aroma, and texture. The superior performance of the 30% MPPS formulation demonstrates that moderate pumpkin seed incorporation provides functional and nutritional benefits while preserving the desirable sensory characteristics of PS. This finding is particularly important because it identifies a substitution level that improves product functionality without compromising sensory acceptance, thereby supporting the development of value-added nut-based spreads with enhanced nutritional quality.

**Table 7:** Sensory acceptance scores of pistachio spreads containing different levels of pumpkin seed paste (*n* = 15 assessors).

Treatment	Color	Odor	Taste	Total acceptance
10%	3.6ᵃ	3.7ᵃ	3.7ᵃ	3.7ᵃ
20%	3.5ᵃ	3.8ᵃ	3.6ᵃ	3.7ᵃ
30%	3.5ᵃ	3.8ᵃ	3.8ᵃ	3.8ᵃ
40%	2.6ᵇ	2.6ᵇ	3.0ᵇ	3.0ᵇ
50%	2.5ᵇ	2.6ᵇ	2.6ᵇ	2.6ᵇ

Different superscript letters within the same column indicate significant differences according to one-way ANOVA followed by Tukey's multiple comparison test (*p* < 0.05).

These findings are in agreement with previous studies that examined the sensory impact of incorporating pumpkin seeds or other seed pastes into nut-based spreads. Pal et al. (2026) reported that moderate incorporation of pumpkin seed paste (up to 30%) improved taste and overall acceptability without adversely affecting color or odor in plant-based spreads (Pal et al., 2026). Similarly, Saydam et al. (2025) observed that pistachio spreads enriched with 20–30% pumpkin seed paste achieved the highest sensory scores for flavor and aroma, while higher substitution levels resulted in decreased consumer preference (Dursun Saydam et al., 2025). Shakerardekani, Karim, Ghazali and Chin, 2013, Shakerardekani, Karim, Ghazali and Chin, 2015 reported comparable results in pistachio spreads supplemented with natural seed pastes, emphasizing that moderate inclusion enhanced sensory attributes, whereas excessive levels negatively influenced flavor balance and consumer acceptance (Ahmad Shakerardekani et al., 2013; Ahmad Shakerardekani et al., 2015). Collectively, these studies corroborate the present observation that a 30% incorporation of pumpkin seed paste provides an optimal balance between improved sensory quality and maintenance of characteristic PS properties.

#### 3.4.2 Colorimetric properties

Instrumental color analysis supported the sensory findings regarding the effect of pumpkin seed paste incorporation on product appearance. Up to 30% substitution, no significant changes were observed in lightness (L*), while slight increases in redness (a*) and yellowness (b*) were attributed to the natural pigments present in pumpkin seed paste. At higher substitution levels (40–50%), more pronounced changes in color parameters, particularly increased lightness, were detected and coincided with lower sensory color acceptance. Considering both instrumental and sensory evaluations, the formulation containing 30% pumpkin seed paste was identified as optimal, providing desirable color characteristics and the highest overall acceptance. These results indicate that moderate incorporation of pumpkin seed paste can improve product functionality without negatively affecting visual quality, whereas excessive substitution may reduce consumer preference. Pal et al. (2026) reported that pumpkin seed spreads exhibited moderate changes in L*, a*, and b* values at low-to-moderate substitution levels, with sensory evaluations confirming acceptable color and overall appearance (Pal et al., 2026). Saydam et al. (2025) observed that pumpkin seed addition slightly increased redness (a*) and yellowness (b*) in nut-based spreads, which correlated with positive consumer acceptance (Dursun Saydam et al., 2025). Similarly, Shakerardekani, Karim, Ghazali and Chin, 2013, Shakerardekani, Karim, Ghazali and Chin, 2015 reported that pistachio spreads enriched with natural seed pastes showed minor but perceptible color changes, which remained within acceptable sensory limits (Ahmad Shakerardekani, Karim, Ghazali and Chin, 2013, Shakerardekani, Karim, Ghazali and Chin, 2015). Overall, these studies corroborate the present finding that moderate incorporation of pumpkin seed paste (up to 30%) maintains desirable color characteristics while enhancing nutritional and functional properties.

### 3.5 Quality evaluation of the optimized sample

#### 3.5.1 Oxidative stability

Table 8 summarizes the changes in peroxide value (PV) and acidity of MPPS and PS during 90 days of storage at 25 °C. Both parameters increased progressively throughout storage, confirming the occurrence of lipid oxidation and hydrolytic degradation in both formulations. However, MPPS consistently exhibited lower PV and acidity than PS, indicating improved oxidative stability following partial substitution of pistachio paste with pumpkin seed paste. The slower increase in PV observed in MPPS suggests that pumpkin seed incorporation delayed the initiation and propagation of lipid oxidation. This improvement may be associated with the naturally occurring antioxidant compounds present in pumpkin seeds, including tocopherols, phenolic compounds, and other bioactive constituents that are capable of scavenging free radicals and interrupting lipid peroxidation reactions. Furthermore, differences in the physical structure of the spread, such as improved emulsion stability and reduced oxygen diffusion within the matrix, may also have contributed to the lower oxidation rate. Nevertheless, these mechanisms cannot be conclusively confirmed in the present study because individual antioxidant compounds and microstructural characteristics were not directly determined. Therefore, the proposed explanations should be regarded as potential mechanisms rather than definitive evidence. The higher DPPH radical scavenging activity observed for MPPS supports the hypothesis that enhanced antioxidant capacity contributed to its improved oxidative stability. Although DPPH activity does not directly quantify lipid oxidation in food systems, the consistency between antioxidant activity and lower peroxide formation suggests that the incorporation of pumpkin seed paste improved the resistance of the formulation to oxidative deterioration during storage. Peroxide value and acidity were selected because they are internationally recognized quality indices for monitoring the primary stages of lipid oxidation and hydrolytic rancidity in fat-rich foods and are widely applied for shelf-life evaluation of edible oils and nut-based products. However, these parameters mainly reflect early oxidative changes and do not provide information on secondary oxidation products or volatile compounds responsible for off-flavor development. Consequently, the oxidative stability assessment presented here should be interpreted within this limitation. Future studies should incorporate complementary analytical techniques, such as anisidine value, TBARS, volatile compound analysis (GC–MS), or oxidation induction measurements (e.g., Rancimat or Oxitest), to provide a more comprehensive evaluation of oxidation mechanisms. Overall, the findings demonstrate that partial replacement of pistachio paste with pumpkin seed paste improved the oxidative stability of the spread during storage. Beyond enhancing antioxidant capacity, the results suggest that formulation-induced modifications in the physical organization of the lipid phase may also influence oxidation behavior. This highlights the importance of considering both chemical composition and structural characteristics when developing shelf-stable nut-based spreads.These findings are in agreement with previous reports, including those of (Dini et al., 2025; Farrokhi et al., 2025; Ahmad Shakerardekani et al., 2015), who also observed comparable radical scavenging activity in pistachio-based systems.

**Table 8:** Changes in oxidative stability parameters of PS and MPPS during 90 days of storage at 25 °C.

Time (day)	MPPS		PS	
	**Peroxide (meq/kg)**	**Acidity (%)**	**Peroxide (meq/kg)**	**Acidity (%)**
0	0.12^f^	0.18^d^	0.65^d^	0.38^e^
15	0.22^e^	0.22^c^	0.85^c^	0.51^d^
30	0.46^d^	0.26^b^	0.98^c^	0.67^c^
45	1.40^c^	0.29^b^	1.50^b^	0.70^bc^
60	1.58^b^	0.33^a^	1.62^b^	0.75^b^
90	1.86^a^	0.36^a^	2.00^a^	0.96^a^

Different superscript letters within the same column indicate significant differences according to one-way ANOVA followed by Tukey's multiple comparison test (*p* < 0.05).

#### 3.5.2 Physical stability

Table 9 compares the oil separation of MPPS and PS during storage as an indicator of physical stability. Oil separation increased progressively in both formulations, reflecting the structural relaxation that commonly occurs in concentrated fat-based dispersions during storage. However, MPPS consistently exhibited lower oil separation than PS, indicating that the incorporation of pumpkin seed paste improved the physical stability of the spread. The enhanced stability of MPPS is likely associated with the compositional characteristics of pumpkin seed paste. Pumpkin seeds contain proteins, phospholipids, and other naturally occurring amphiphilic compounds that can enhance fat binding, stabilize the dispersed lipid phase, and strengthen the structural network of the spread. In addition, the higher protein content and insoluble solids present in pumpkin seed paste may increase matrix viscosity and reduce lipid mobility, thereby limiting oil migration and phase separation during storage. These mechanisms are consistent with the behavior of concentrated suspensions, in which stronger particle–particle and particle–fat interactions contribute to improved structural integrity and reduced syneresis. Although these mechanisms provide a plausible explanation for the observed improvement in physical stability, they were not directly investigated in the present study. Microstructural analyses, such as confocal laser scanning microscopy or scanning electron microscopy, together with rheological characterization, would be required to confirm the influence of pumpkin seed components on emulsion structure and oil immobilization.

**Table 9:** Comparison of oil separation in PS and MPPS during 90 days of storage.

Time (day)	MPPS	PS
	**Oil separation (mL/200 g)**	**Oil separation (mL/200 g)**
15	2.37^b^	3.45^a^
30	2.50^b^	3.70^a^
45	2.79^b^	3.92^a^
60	3.01^b^	4.27^a^
90	3.21^b^	4.60^a^

Different superscript letters within the same row indicate significant differences according *t-*test (*p* < 0.05).

Overall, the results demonstrate that partial substitution of pistachio paste with pumpkin seed paste improved the physical stability of the spread by reducing oil separation during storage. This improvement is technologically important because lower oil separation may enhance product appearance, spreadability, and acceptance while reducing the need for additional stabilizing additives. Consequently, pumpkin seed paste represents a promising natural ingredient for improving the physical stability and quality of nut-based spreads.

Ahmad Shakerardekani et al. (2013) demonstrated that the incorporation of suitable emulsifiers and stabilizers in pistachio spread significantly reduced oil separation and improved structural consistency over storage, highlighting the importance of protein–lipid interactions and fat-binding capacity in stabilizing high-fat systems (Ahmad Shakerardekani et al., 2013). Likewise, Emadzadeh et al. (2013) reported that optimizing the ratio of nuts and added oils through response surface methodology effectively minimized oil migration and improved storage stability in pistachio spread formulations (Emadzadeh et al., 2013). Mousazadeh et al. (2014) further confirmed that balanced proportions of solid particles and lipid phase contribute to enhanced viscosity and reduced phase separation, resulting in improved physical stability of pistachio spread. In addition, studies on other seed- and nut-based spreads have shown comparable trends (Mousazadeh et al., 2014). Similarly, (MCCLEMENTS & DECKER Mcclements & Decker, 2000) emphasized that increasing the solid matrix strength and improving emulsification efficiency significantly decreases gravitational oil separation in fat-rich dispersions (MCCLEMENTS & DECKER Mcclements & Decker, 2000). Collectively, these studies indicate that enhanced protein content, improved emulsification, and strengthened solid–fat interactions may contribute to limiting oil mobility and improving the physical stability of plant-based spreads during storage. In particular, heat-assisted pH-shifting of pumpkin seed protein isolate enhanced its emulsifying performance and promoted the adsorption of protein-based particles onto oil droplets, resulting in improved emulsion stability during storage (Sun et al., 2024).

#### 3.5.3 Determination of water activity

Table 10 presents the changes in water activity (aw) of MPPS during storage at 25 °C. A gradual decline in aw was observed throughout the storage period, indicating progressive changes in water distribution within the spread matrix. Despite this reduction, aw remained well below the threshold generally required for the growth of most bacteria, suggesting that the formulation maintained a low potential for microbial growth under ambient storage conditions. The decrease in aw is likely associated with moisture redistribution and stronger immobilization of water molecules within the product matrix. The relatively high sugar content, together with the proteins and dietary fiber contributed by pumpkin seed paste, may have enhanced water binding and reduced the amount of free water available for microbial growth. In addition, structural rearrangements occurring during storage could further restrict water mobility by increasing interactions between water molecules and macromolecular components of the spread.

**Table 10:** Measurement of water activity of the optimal sample at 25 °C during 3 months of storage.

aw	Time (day)
0.446	0
0.440	15
0.415	30
0.407	45
0.392	60
0.345	90

From a shelf-life perspective, the maintenance of low aw complements the observed improvements in oxidative and physical stability, indicating that the optimized formulation possesses multiple factors contributing to product preservation. Nevertheless, water activity alone cannot fully predict microbiological stability because microbial growth is also influenced by factors such as pH, processing conditions, packaging, and storage environment. Therefore, although the low aw values strongly suggest favorable conditions for microbiological stability, microbiological analyses would be required to confirm product microbiological quality and safety throughout storage. Overall, the results demonstrate that the optimized MPPS maintained low water activity during storage, supporting its suitability as a shelf-stable nut-based spread. The combination of reduced water availability, improved oxidative stability, and enhanced physical stability provides evidence of the formulation's potential for extended storage stability under ambient conditions.

Recent studies on nut-and seed-based spreads have confirmed that low water activity is a primary factor governing microbial stability and shelf life in high-fat systems. Kılıç et al. (2025) reported that pistachio spread formulations exhibited aw values below 0.50, which remained stable during storage and effectively prevented microbial proliferation under ambient conditions (Kılıç et al., 2025). Similarly, Malvano et al. (2024) demonstrated that high-protein PS formulated through mixture design maintained aw values well below the microbial growth threshold (<0.60), ensuring microbiological safety and extended shelf life (Malvano et al., 2024). In addition, Peng et al. (2021) observed that roasted pumpkin seed products exhibited low water activity due to their high lipid content and limited free moisture, contributing to improved storage stability (Peng et al., 2021). Collectively, these recent findings confirm that maintaining aw values well below 0.60 in nut- and seed-based spreads is strongly associated with microbial safety, structural stability, and prolonged shelf life under ambient storage conditions.

#### 3.5.4 Textural properties

The results presented in Table 11 demonstrate that storage time significantly influenced both spreadability and hardness in PS and MPPS. In terms of spreadability, both samples showed a decreasing trend over time. Specifically, the spreadability of MPPS decreased from 0.035 at day 0 to 0.030 at day 90, while PS exhibited a reduction from 0.027 to 0.022 over the same period. Despite this decline, MPPS consistently maintained higher spreadability compared to PS throughout storage, indicating a softer and more easily spreadable texture. Conversely, hardness increased progressively in both formulations during storage. The hardness of MPPS increased from 0.30 to 0.36, whereas PS showed a more pronounced increase from 0.35 to 0.45. The higher hardness values observed in PS suggest a firmer structure compared to MPPS. Notably, MPPS exhibited lower hardness values at all time points, which is consistent with its higher spreadability.The observed increase in hardness and decrease in spreadability over time can be attributed to structural changes in the lipid matrix, including fat crystallization and possible moisture redistribution. The improved textural properties of MPPS may be related to the presence of bioactive compounds in pumpkin seeds, such as phospholipids and natural emulsifiers, which can contribute to matrix stability and reduce structural rigidity. Overall, the incorporation of pumpkin seed paste resulted in MPPS having superior spreadability and lower hardness compared to PS, indicating improved textural quality and potentially greater consumer acceptability during storage. Kılıç et al. (2025) reported that increasing the proportion of non-fat solids and optimizing emulsifier levels in pistachio spread significantly reduced hardness and improved spreadability, while maintaining structural cohesiveness (Kılıç et al., 2025). Their results also showed that textural parameters were strongly dependent on fat–solid interactions and could be effectively controlled through formulation adjustments. Similarly, Malvano et al. (2024) observed that high-protein pistachio spreads formulated via mixture design exhibited lower hardness and enhanced spreadability when protein-rich components were incorporated, which was attributed to improved matrix integration and modified lipid distribution (Malvano et al., 2024). The authors further reported that spreads containing balanced protein–lipid ratios maintained better textural stability during storage, with slower increases in hardness over time. In addition, Ahmad Shakerardekani et al. (2013) demonstrated that emulsifier incorporation and ingredient ratio optimization in pistachio spread significantly affected rheological and textural behavior, particularly adhesiveness and firmness. Their findings indicated that improved dispersion of the fat phase reduced structural rigidity and enhanced consumer-perceived spreadability (Ahmad Shakerardekani et al., 2013). Collectively, these studies support the importance of compositional balance, protein–lipid interactions, and matrix structuring mechanisms in determining hardness and spreadability in nut- and seed-based spreads.

**Table 11:** Textural properties of PS and MPPS with 30% pumpkin seed replacement during 90 days of storage.

Time (day)	Hardness – PS	Hardness – MPPS	Spreadability – PS	Spreadability – MPPS
0	0.027	0.035	0.35	0.30
30	0.027	0.036	0.38	0.31
60	0.025	0.033	0.41	0.34
90	0.022	0.030	0.45	0.36

#### 3.5.5 Rheological properties

The rheological behavior of the optimized formulation was successfully described by the Power Law model, demonstrating a strong relationship between shear rate and flow response. The obtained parameters indicate that the product exhibits pronounced shear-thinning behavior, which is a desirable characteristic for nut-based spreads. This pseudoplastic behavior allows the apparent viscosity of the spread to decrease during application, facilitating easy spreading, while maintaining sufficient structural integrity under resting conditions to prevent excessive flow and phase separation. The difference between the ascending and descending flow curves confirmed the presence of thixotropic behavior, indicating that the internal structure of the spread undergoes partial breakdown during shearing and gradually recovers after the removal of stress. This structural reversibility is technologically important because it provides a balance between processability and stability. In practical applications, a thixotropic spread can flow easily during spreading while recovering enough structure afterward to maintain product appearance and potentially minimize oil migration.

The time-dependent rheological response further demonstrated that the structural changes occurring under constant shear can be described by a first-order stress decay model with a non-zero equilibrium stress. This behavior indicates that the product structure is not completely disrupted during shear application; instead, a portion of the internal network remains intact. Such behavior is typical of semi-solid food systems in which a continuous lipid phase interacts with dispersed solid particles, proteins, and other structural components. The gradual decrease in shear stress during shearing suggests progressive rearrangement of the internal matrix rather than rapid structural collapse. This indicates that the formulation possesses adequate structural strength while remaining sufficiently shear-sensitive for consumer handling. The observed rheological characteristics are particularly beneficial for PS because they may contribute to desirable sensory attributes, including smooth mouthfeel and controlled spreadability, as well as structural stability during storage and consumption.

Overall, the rheological analysis confirms that the optimized formulation exhibits a well-balanced structure characterized by shear-thinning flow, thixotropic recovery, and controlled structural breakdown. These properties support its suitability as a spreadable product with desirable processing performance and potentially favorable consumer acceptance. Similarly, Malvano et al. (2024) demonstrated that high-protein nut spreads with balanced fat–protein ratios showed pronounced thixotropic behavior and controlled structural decay, allowing easy application without oiling off (Malvano et al., 2024). Collectively, these studies support the present results, indicating that careful formulation of pistachio and pumpkin seed components can yield spreads with favorable shear-thinning, thixotropic, and structural recovery characteristics suitable for both processing and consumption. Principato et al. (2024) reported pseudoplastic behavior across the investigated spreads and showed that differences in apparent viscosity and flow behavior were associated with textural and oral sensory characteristics, including perceptions of fluidity, stickiness, and particle-related mouthfeel (Principato et al., 2024).

#### 3.5.6 Determination of oxidation kinetic parameters

Lipid oxidation is one of the major factors limiting the shelf life of high-fat products such as PS, where the high content of unsaturated fatty acids increases susceptibility to oxidative deterioration. The effect of storage temperature on hydroperoxide formation was evaluated using kinetic modeling under different storage conditions. The experimental oxidation data were fitted to zero-, first-, and second-order kinetic models, and the results indicated that the zero-order model provided the most appropriate description of peroxide formation during storage. The suitability of the zero-order model suggests that hydroperoxide accumulation occurred at an approximately constant rate within the studied storage period, indicating that the concentration of reactive substrates remained sufficiently high and did not represent a limiting factor during the early stages of oxidation. This behavior is commonly observed in lipid-rich food systems, where oxygen availability and temperature are dominant factors controlling the oxidation rate rather than the depletion of oxidizable compounds. The oxidation rate increased markedly with increasing storage temperature, demonstrating the strong thermal sensitivity of lipid oxidation reactions. The higher oxidation rate observed at elevated temperatures can be attributed to increased molecular mobility, enhanced oxygen diffusion, and accelerated formation of free radicals involved in lipid peroxidation pathways. In contrast, refrigerated storage reduced oxidative progression by limiting the kinetic energy required for initiation and propagation reactions. Although alternative kinetic models also provided acceptable statistical fits, the superior performance of the zero-order model indicates that it more accurately represents peroxide accumulation in this system. This finding provides valuable information for predicting shelf-life behavior and selecting appropriate storage conditions for PS-based products. From a practical perspective, the strong temperature dependency of oxidation emphasizes the importance of temperature control during storage and distribution. Maintaining lower storage temperatures can substantially delay hydroperoxide formation and preserve product quality by reducing the rate of primary oxidation reactions. Overall, the kinetic analysis provides a mechanistic understanding of oxidative deterioration in PS and offers a useful approach for estimating storage stability and designing strategies to improve shelf life. The observed temperature-dependent zero-order kinetics of hydroperoxide formation in pistachio oil align with previous reports on lipid oxidation in high-fat nut products. Similar findings have demonstrated that pistachio and other tree nut oils exhibit zero-order oxidation behavior under controlled storage conditions, with reaction rates strongly increasing with temperature (Shahidi & Zhong, 2010). Likewise, refrigerated storage has been shown to significantly slow peroxide formation in nut oils, whereas elevated temperatures accelerate oxidative deterioration and reduce shelf life (Tavakolipour et al., 2017). Collectively, these studies corroborate the present results, confirming that careful control of storage temperature is critical for preserving the chemical stability of PS and MPPS. The zero-order kinetic model provided the best description of peroxide value development in MPPS during storage. The zero-order rate constants (k₀) were 0.047, 0.148, and 0.3688 meq O₂ kg^−1^ day^−1^ at 4, 25, and 40 °C, respectively, while the corresponding initial peroxide values (PV₀) were 7.315, 4.756, and 2.878 meq O₂/kg (Table 12). The increase in k₀ with temperature indicates a substantial acceleration of peroxide formation at elevated temperature.

**Table 12:** Kinetic Parameters of Lipid Oxidation in MPPS at Different Temperatures.

Temperature	Zero-order reaction			First-order reaction			Second-order reaction		
	**C₀ (meq/kg)**	**R₀₂**	**K₀ (meq**/**kg** **day**^**−1**^**)**	**C₁ (meq/kg)**	**R₁₂**	**K₁ (day**^**−1**^**)**	**C₂ (meq/kg)**	**R₂₂**	**K₂ (kg meq**^**−1**^ **day**^**−1**^**)**
4°C	7.315	0.95	0.047	7.88	0.98	0.005	8.04	0.90	0.0005
25 °C	4.756	0.97	0.148	5.07	0.93	0.023	5.51	0.85	0.002
40 °C	2.878	0.97	0.368	5.75	0.98	0.013	6.10	0.95	0.0013

The kinetic and thermodynamic parameters associated with lipid oxidation in MPPS at different storage temperatures are presented in Table 13. These parameters, including time to 50% increase in PV (t₅₀), activation energy (Ea), Gibbs free energy of activation (ΔG‡), enthalpy of activation (ΔH‡), and entropy of activation (ΔS‡), provide valuable information regarding the temperature dependence of oxidation reactions and the oxidative stability of the product during storage.

**Table 13:** Kinetic and thermodynamic parameters of lipid oxidation in MPPS at different storage temperatures.

Storage temp. (°C)	∆G‡ (KJ/mol)	∆S‡ (KJ/mol/K)	∆H‡ (KJ/mol)	Ea (KJ/mol)	t₅₀ (day)
4	74.82	−0.131 38.43 40.87			77.65
25	77.58				16.07
40	79.55				3.91

The kinetic and thermodynamic parameters indicated a strong influence of storage temperature on the oxidative stability of MPPS. As storage temperature increased from 4 to 40 °C, the time to 50% increase in PV (t₅₀) decreased markedly from 77.65 to 3.91 days, demonstrating a substantial acceleration of peroxide formation and lipid oxidation at elevated temperatures. This behavior confirms the temperature dependence of oxidative reactions and highlights the importance of low-temperature storage for maintaining product quality. The Gibbs free energy of activation (ΔG‡) increased from 74.82 to 79.55 kJ/mol with increasing temperature. The relatively high ΔG‡ values observed in the present study suggest that the oxidation process in MPPS is kinetically hindered, which may be attributed to the presence of natural antioxidants from pumpkin seed paste. Similar observations have been reported for lipid-rich food systems, where higher ΔG‡ values were associated with greater oxidative resistance and improved storage stability (Tolve et al., 2022). The zero-order rate constant increased markedly with increasing storage temperature. Arrhenius analysis of the k₀ values resulted in an activation energy (Ea) of 40.87 kJ/mol, confirming the pronounced temperature dependence of lipid oxidation in MPPS. This value indicates a moderate energy barrier for peroxide formation and suggests that oxidation proceeds more rapidly as temperature increases. Comparable activation energy values have been reported for oil- and fat-based food systems, where lipid oxidation rates increased significantly with temperature due to enhanced molecular mobility and oxygen diffusion (Kaseke et al., 2021). The positive enthalpy of activation (ΔH‡ = 38.43 kJ/mol) indicates that the oxidation process is endothermic and requires energy to form the activated complex. Similar positive ΔH‡ values have been reported in studies of lipid oxidation in food emulsions and spreads, suggesting that thermal energy promotes the formation of oxidation intermediates and hydroperoxides (Tolve et al., 2022). The negative entropy of activation (ΔS‡ = −0.131 kJ/mol·K) suggests that the activated complex is more ordered than the reactants. This reduction in molecular randomness during the transition state is characteristic of oxidation reactions involving the formation of organized intermediate complexes. Negative ΔS‡ values have also been reported in thermodynamic studies of lipid oxidation, indicating increased molecular organization during the rate-determining step of the reaction (Kaseke et al., 2021).

## 4 Conclusion

The present study demonstrates that partial substitution of pistachio paste with pumpkin seed paste represents an effective strategy for improving the quality and storage stability of pistachio-based spreads. The optimized MPPS, containing 30% pumpkin seed paste, exhibited enhanced oxidative stability, improved physical stability, and favorable sensory characteristics while maintaining acceptable textural properties throughout storage. Kinetic analysis provided quantitative information on the temperature dependence of peroxide formation and the oxidative stability of MPPS under the studied storage conditions. The marked reduction in t₅₀ with increasing temperature further demonstrated the strong temperature dependence of peroxide formation. From a scientific perspective, this work provides new insight into the combined effects of pumpkin seed incorporation on the sensory, physicochemical, rheological, and kinetic characteristics of pistachio spread. The integration of formulation optimization with oxidation kinetics offers a comprehensive approach for designing stable plant-based spreads and contributes to the limited knowledge on the thermodynamic and kinetic behavior of composite nut products. From a practical standpoint, the optimized MPPS formulation offers the food industry a cost-effective alternative to conventional pistachio spread by partially replacing pistachio with pumpkin seed while maintaining favorable sensory acceptance scores and improving product stability. The enhanced oxidative resistance may also reduce quality deterioration during storage and may contribute to extending the oxidative stability and potential storage life of the product, supporting the development of value-added functional spreads with improved market potential. Future studies should investigate the influence of different pumpkin seed cultivars and roasting conditions on product quality, evaluate the bioaccessibility of antioxidant compounds during digestion, and validate shelf-life predictions under commercial storage conditions. In addition, incorporating other functional ingredients or natural antioxidants and conducting acceptance studies on a larger scale would further support the industrial application of mixed nut- and seed-based spreads.

## CRediT authorship contribution statement

**Arian Nahalkar:** Writing – original draft. **Mostafa Shahidi-Noghabi:** Writing – review & editing, Supervision, Data curation, Conceptualization. **Sara Naji-Tabasi:** Writing – review & editing, Validation, Supervision.

## Declaration of competing interest

The authors declare that they have no known competing financial interests or personal relationships that could have appeared to influence the work reported in this paper.

## Data Availability

Data will be made available on request.
